# Development and validation of CYP26A1 inhibition assay for high-throughput screening

**DOI:** 10.1002/biot.202300659

**Published:** 2024-06

**Authors:** Srilatha Sakamuru, Dongping Ma, Jocylin D. Pierro, Nancy C. Baker, Nicole Kleinstreuer, James J. Cali, Thomas B. Knudsen, Menghang Xia

**Affiliations:** 1 Division of Pre-clinical Innovation, National Center for Advancing Translational Sciences, National Institutes of Health, Rockville, Maryland, USA; 2 Promega Corporation, Madison, Wisconsin, USA; 3 Center for Computational Toxicology and Exposure, Office of Research and Development, United States Environmental Protection Agency, Research Triangle Park, North Carolina, USA; 4 Leidos, Research Triangle Park, North Carolina, USA; 5 National Toxicology Program Interagency Center for the Evaluation of Alternative Toxicological Methods, National Institute of Environmental Health Sciences, National Institutes of Health, Research Triangle Park, North Carolina, USA

**Keywords:** all-*trans* retinoic acid (atRA), CYP26, cytochrome P450 (CYP), retinoic acid receptor (RAR)

## Abstract

All-*trans* retinoic acid (atRA) is an endogenous ligand of the retinoic acid receptors, which heterodimerize with retinoid X receptors. AtRA is generated in tissues from vitamin A (retinol) metabolism to form a paracrine signal and is locally degraded by cytochrome P450 family 26 (CYP26) enzymes. The CYP26 family consists of three subtypes: A1, B1, and C1, which are differentially expressed during development. This study aims to develop and validate a high throughput screening assay to identify CYP26A1 inhibitors in a cell-free system using a luminescent P450-Glo assay technology. The assay performed well with a signal to background ratio of 25.7, a coefficient of variation of 8.9%, and a Z-factor of 0.7. To validate the assay, we tested a subset of 39 compounds that included known CYP26 inhibitors and retinoids, as well as positive and negative control compounds selected from the literature and/or the ToxCast/Tox21 portfolio. Known CYP26A1 inhibitors were confirmed, and predicted CYP26A1 inhibitors, such as chlorothalonil, prochloraz, and SSR126768, were identified, demonstrating the reliability and robustness of the assay. Given the general importance of atRA as a morphogenetic signal and the localized expression of *Cyp26a1* in embryonic tissues, a validated CYP26A1 assay has important implications for evaluating the potential developmental toxicity of chemicals.

## Lay Summary

All-trans retinoic acid (atRA) is essential for embryonic development and tissue homeostasis in adults. The atRA is locally generated from retinol metabolism and primarily degraded by cytochrome p450 family 26 (CYP26) enzymes. Here, we developed and validated an enzyme-based CYP26A1 high-throughput assay that can be used to identify CYP26A1 inhibitors by screening the chemical libraries.

## Graphical Abstract

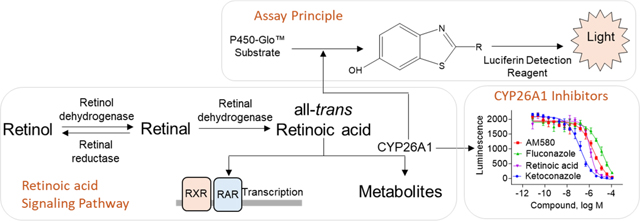

## 1 | INTRODUCTION

**FIGURE 1:**
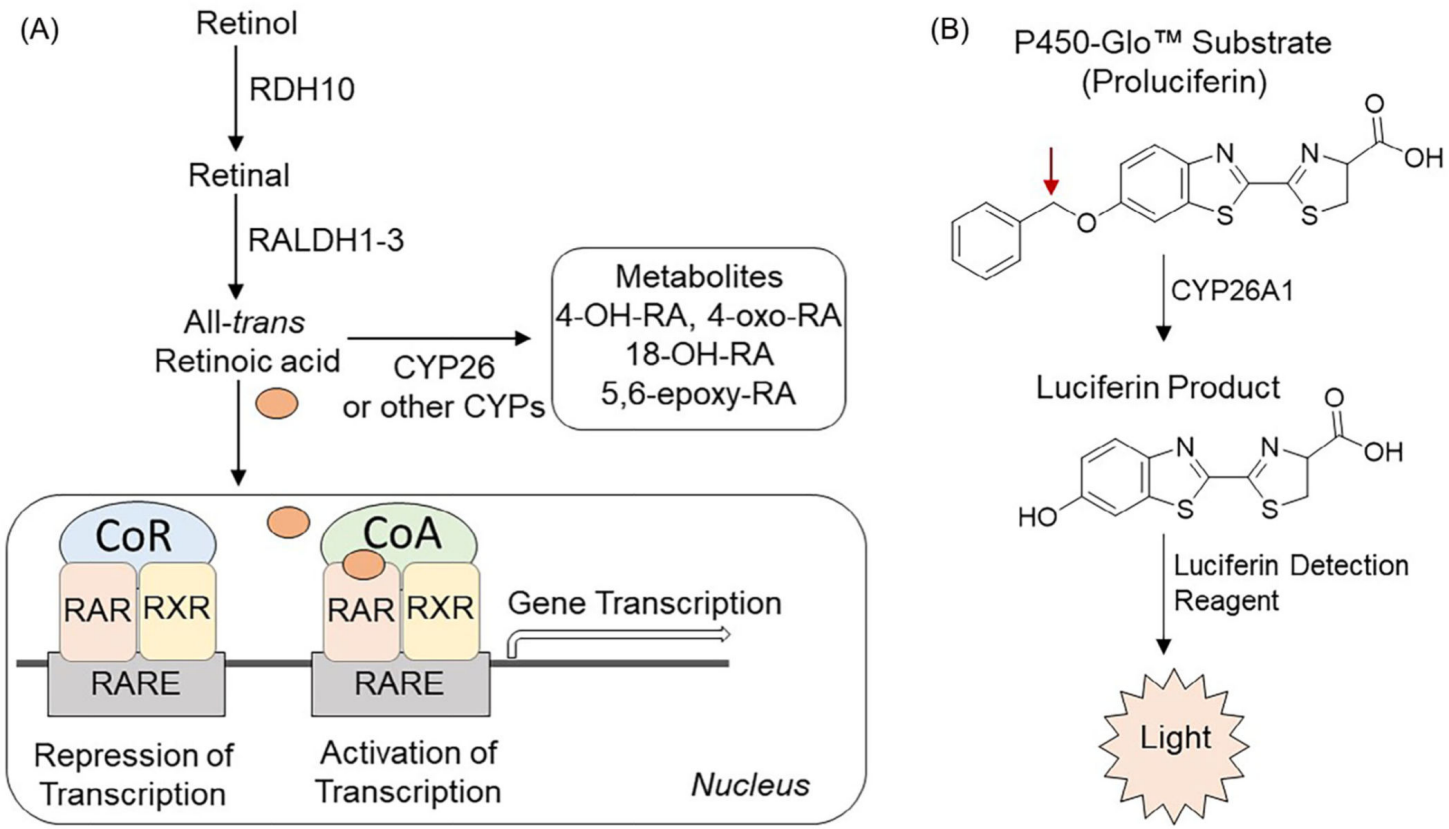
(A) General scheme of retinoic acid signaling pathway and metabolism by CYP26 enzymes. Retinol is converted to all-*trans* retinoic acid (atRA) via two oxidative steps, first to retinal mainly by retinol dehydrogenase 10 (RDH10) and then to atRA by retinal dehydrogenases 1 to 3 (RALDH1–3). atRA is further metabolized by the CYP26 and other CYP family of enzymes to primary metabolites, including 4-OH-RA, 18-OH-RA, and 5,6-epoxy-RA and are further metabolized to polar metabolites such as 4-oxo-RA. atRA is the activating ligand for the retinoic acid receptors (RARs), which heterodimerize with retinoid X receptors (RXRs) on the retinoic acid response element (RARE). In the inactive state, nuclear receptor co-repressor (CoR) complexes tighten and prevent the transcription of genes. Recruitment of co-activator (CoA) promotes transcription of genes downstream of the RARE; (B) Conversion of P450-Glo^™^ substrate by CYP26A1. CYP enzyme acts on a luminogenic P450-Glo^™^ substrate (first reaction) to produce a luciferin product that generates light with the luciferin detection reagent (second reaction). The amount of light produced is proportional to CYP activity. The red arrow indicates the site of modification by CYP enzyme.

All-*trans* retinoic acid (atRA), an active metabolite of vitamin A (retinol), plays a crucial role in regulating cell growth and differentiation in many tissues.^[1]^ It acts as an endogenous ligand, along with its isomer, 9-*cis*-RA, for two families of ligand-activated nuclear receptors: retinoic acid receptors (RAR*α*, *β*, and *γ*) and retinoid X receptors (RXR*α*, *β*, and *γ*), respectively. RAR/RXR heterodimers, together with co-activators or co-repressors, regulate gene expression through retinoic acid response elements (RARE) in many genes that function in metabolic and developmental pathways (Figure 1A).^[2,3]^ The atRA signaling pathway plays a crucial role in developmental processes and maintenance of cellular phenotype.^[4]^ Disrupting the atRA signaling pathway can lead to altered retinoid concentrations in target tissues, resulting in heightened or diminished RAR-mediated signaling. The main pathway for atRA elimination occurs through oxidative metabolism via 4-hydroxylation, which is mediated by a range of cytochrome P450 enzymes (CYP).^[3,5]^

The first CYP to be identified as a major contributor to atRA 4-hydroxylation in human liver was CYP2C8, with CYP3A subfamily as minor participants.^[6]^ Several other human CYPs were subsequently shown to catalyze 4-hydroxylation of atRA, including CYP1A1, CYP1A2, CYP2C9, CYP3A4, CYP3A7, and CYP26.^[3]^ The three isoforms of CYP26 family, CYP26A1, CYP26B1, and CYP26C1 are considered the primary mammalian atRA hydroxylases.^[7,8]^ The primary atRA metabolites include 4-OH-RA, 18-OH-RA, and 5,6-epoxy-RA, among which 4-OH-RA appeared to be the most common metabolite formed by CYP26, which can then be further metabolized to polar secondary metabolites such as diols and 4-oxo-RA.^[9,10]^ All three isoforms are subject to cell-specific regulation in developing and adult tissues.^[11]^ CYP26A1 is enriched in the human liver and is mainly responsible for the hepatic clearance of atRA.^[12]^ CYP26B1 is enriched in placenta, ovary, testes, and intestine, while CYP26C1 is enriched in brain and liver.^[9]^ Knockout of these three genes was shown to be severely detrimental^[9]^; a post-natal inducible global knockout of *Cyp26a1* and *Cyp26b1* in adult mice or juvenile resulted in severe dermatitis, hyperkeratosis and hyperplasia, blepharitis, splenomegaly, and inflammation,^[13]^ whereas the loss of both *Cyp26a1* and *Cyp26c1* in mice blocked the production of cranial neural crest cells in the forebrain and midbrain.^[14]^ As such, the identification of CYP26-active inhibitors will have significant implications for developmental and homeostatic processes and therapeutic intervention.^[15–17]^

Currently, there is no in vitro CYP26A1 inhibition assay available in a high-throughput screening format. Furthermore, the Organization of Economic Cooperation and Development (OECD) in their detailed 2021 review of the retinoid system emphasized the development of a CYP26 inhibition assay as a high priority [OECD, Detailed review paper on the retinoid system Series on Testing and Assessment, (2021) No. 343 https://one.oecd.org/document/ENV/CBC/MONO(2021)20/en/pdfref]. Here, we developed a high-throughput enzyme-based CYP26A1 assay. The assay relies on a probe substrate that engages the enzyme active site and is converted to a readily detected product (e.g., via an optical property). This probe-based approach has been widely used and validated within the broad family of cytochrome p450 enzymes to identify and characterize inhibitors of various mechanisms, including substrates that compete for the active site.^[18–20]^ We validated the assay against a subset of 39 compounds, which included known CYP26 inhibitors and retinoids, as well as positive and negative control compounds selected from the literature and/or the ToxCast/Tox21 portfolio. The compounds’ activities on CYP26A1 enzyme were then compared with their effects in CYP26B1, CYP1A2, CYP3A4, CYP2C9, CYP2C19, CYP2D6, RAR, and RXR in vitro assays to evaluate their selectivity toward CYP26A1. The successful development and validation of a high-throughput screening assay for CYP26A1 enzyme activity reported here paves the way for identifying novel CYP26A1 inhibitors from chemical libraries.

## 2 | MATERIALS AND METHODS

### 2.1 | Chemicals

The following chemicals were purchased from Sigma-Aldrich (St. Louis, MO): 9-*cis*-retinoic acid (9-*cis*-RA), 13-*cis*-retinoic acid (13-*cis*-RA), adapalene, aldrin, all-*trans* retinoic acid (atRA), AM580, bensulide, bexarotene, buprofezin, caffeine, captafol, CD437, chlorothalonil, coumaphos, decanal, deltamethrin, dieldrin, endosulfan, endrin, fluconazole, furafylline, indole-3-acetic acid, ketoconazole, oxadiazon, oxalic acid, prochloraz, propargite, quinidine, R115866, raloxifene hydrochloride, retinol, SR 11237, sulfaphenazole, tamibarotene, tazarotene, tazarotenic acid, triadimefon, tributyltin benzoate, and TTNPB. Endosulfan I was purchased from Cayman Chemical (Ann Arbor, MI). Etretinate and tetrabromobisphenol A bis(2-hydroxyethyl) ether (TBBPA-BHEE) were purchased from TCI America (Portland, OR). Fenpyroximate (Z,E) was purchased from Santa Cruz Biotechnology, Inc. (Dallas, TX). Liarozole dihydrochloride was purchased from Tocris Bioscience (Bio-Techne Corporation, Minneapolis, MN). SSR126768 was obtained as a 19 mM stock solution dissolved in DMSO from the compound management at National Center for Advancing Translational Sciences (NCATS). Triflumizole was purchased from Chemservice (West Chester, PA).

### 2.2 | P450-Glo^™^ CYP26A1 assay

P450-Glo^™^ CYP26A1 screening system was purchased as a custom product from Promega Corp. (Madison, WI). The system includes recombinant wild-type human CYP26A1 enzyme co-expressed with P450 oxidoreductase (POR) in *Escherichia coli* expression system and prepared as Bactosomes (purchased from Cypex/BioIVT, Dundee, Scotland, UK; catalog # CYP070), Luciferin-benzyl ether (Luciferin-BE) synthesized as described,^[21]^ KPO4 (pH 7.4) and NADPH regeneration solution consisting of 1.3 mM NADP^+^, 3.3 mM Glucose-6-P, 3.3 mM MgCl_2_, 0.4 U mL^−1^ G-6-P dehydrogenase, and 0.05 mM NaCitrate (concentrations in the final reaction volume). The regenerating system ensures that NADPH remains non-limiting throughout the reaction time course.^[22]^ P450-Glo assays rely on CYP-dependent conversion of a proluciferin probe substrate to D-luciferin that is detected as relative luminescence units (RLU) in a second reaction with luciferase.^[23]^ Two microliters enzyme (0.02 pmol μL^−1^ CYP26A1)- substrate (2 μM Luciferin-BE) mix containing 200 mM KPO4, pH 7.4 was dispensed using a Flying Reagent Dispenser (FRD) BioRaptr 2 (axiVEND, Winter Garden, FL) in white opaque 1536-well medium binding plates (Greiner Bio-One North America Inc., Monroe, NC). The positive control (atRA) along with DMSO-only wells and test compounds were transferred at 23 nL using a Wako Pintool station (Wako Automation, San Diego, CA). Following compound treatment and incubation of the assay plates for 10 min at room temperature, 2 μL NADPH-regeneration solution was added using an FRD to start the reaction. The reaction was continued by incubating the assay plates at 37°C for 30 min. The reaction was stopped by adding 4 μL luciferin detection reagent. The luminescence signal was measured using a ViewLux plate reader (Perkin Elmer, Waltham, MA). The raw data were obtained as relative luminescence units.

### 2.3 | Other P450-Glo^™^ assays

P450-Glo^™^ assays in the current study including CYP26B1, CYP1A2, CYP3A4, CYP2C9, CYP2C19, and CYP2D6 were purchased from Promega Corp.^[23]^ The CYP26B1 assay was a custom assay prepared as described above except it contained recombinant human CYP26B1 co-expressed with POR in *E. coli* (Cypex/BioIVT, Dundee, Scotland, UK; catalog # CYP071) in place of the *Cyp26a1* sequence. Two microliters of various enzyme- substrate mix (i.e., 0.08 pmol μL^−1^ CYP26B1– 50 μM Luciferin-BE; 0.01 pmol μL^−1^ CYP1A2– 100 μM Luciferin-ME; 0.01 pmol μL^−1^ CYP3A4– 25 μM Luciferin-PPXE; 0.01 pmol μL^−1^ CYP2C9– 100 μM Luciferin-H; 0.005 pmol μL^−1^ CYP2C19-10 μM Luciferin-H EGE; or 0.005 pmol μL^−1^ CYP2D6– 30 μM Luciferin-ME EGE) was dispensed using an FRD in white opaque 1536-well medium binding plates. The positive control inhibitor (atRA, furafylline, sulfaphenazole, and quinidine for CYP26B1, CYP1A2, CYP2C9, and CYP2D6 respectively; and ketoconazole for CYP3A4 and CYP2C19) along with DMSO-only wells and test compounds (23 nL) were transferred to assay plates using a Wako Pintool station. Following the compound transfer and incubation of the assay plates for 10 min at room temperature, 2 μL NADPH-regeneration solution was added using an FRD to start the reaction. The reaction was continued by incubating the assay plates for 60 min at 37°C for CYP26B1, CYP2C9, and CYP2C19 assays, or at room temperature for CYP1A2, CYP3A4, and CYP2D6 assays. The reaction was stopped by adding 4 μL luciferin detection reagent. The luminescence signal in each well was measured using a ViewLux plate reader. The raw data were obtained as relative luminescence units.

### 2.4 | RARE-*Luc* reporter gene assay

RARE-*luc* reporter gene cell line, C3RL4 described previously^[24]^ was utilized to identify activators of retinoic acid receptor. C3RL4 is a stable clone derived from mouse multipotent C3H10T1/2 cell line and contains a firefly luciferase reporter gene (*luc*) under the control of the retinoic acid response element (RARE) promoter. C3RL4 cells were cultured in medium consisting of 90% basal medium eagle (BME) (Thermo Fisher Scientific Inc., Waltham, MA) supplemented with 10% heat-inactivated fetal bovine serum (FBS) (Sigma-Aldrich, Inc. St. Louis, MO), 2 mM L-glutamine, 2 μg mL^−1^ puromycin, and 100 U mL^−1^ penicillin – 100 μg mL^−1^ streptomycin (Thermo Fisher Scientific Inc.). The thawing and assay media were same as the culture medium except puromycin addition. The C3RL4 cells were seeded at 1000 in 5 μL assay medium per well in white opaque 1536-well tissue culture plates (Greiner Bio-One North America Inc.) using an FRD. After an overnight incubation of the assay plates at 37°C, 99% humidity, and 5% CO_2_, the positive control (retinol) along with DMSO-only wells and test compounds, were transferred to the assay plates at 23 nL using a Wako Pintool station. The assay plates were incubated with the compounds for 6 h at 37°C, 99% humidity, and 5% CO_2_, followed by the addition of 5 μL One-Glo^™^ luciferase assay reagent (Promega US) using an FRD. After incubation of assay plates for 30 min at room temperature, the luminescence signal was measured using a ViewLux plate reader. The raw data were obtained as relative luminescence units.

### 2.5 | RXR*α*-*Bla* reporter gene assay

GeneBLAzer^®^ RXR*α*-UAS-*bla* HEK293T cell line was purchased from Thermo Fisher Scientific Inc. This cell line contains a human retinoid X receptor alpha (RXR*α*) ligand-binding domain/Gal4 DNA binding domain chimera with a beta-lactamase reporter gene (*bla*) under the control of a UAS response element that is stably integrated into HEK 293T cells. RXR*α*-bla cells were cultured in Dulbecco’s Modified Eagle Medium (DMEM) with GlutaMAX supplemented with 10% dialyzed FBS, 0.1 mM NEAA, 25 mM HEPES, 100 μg mL^−1^ hygromycin, 100 μg mL^−1^ zeocin, and 100 U mL^−1^ penicillin – 100 μg mL^−1^ streptomycin. For thawing cells, culture medium without hygromycin and zeocin was used and the assay medium contains phenol-red free DMEM supplemented with 2% charcoal-stripped FBS, 0.1 mM NEAA, 1 mM sodium pyruvate and 100 U mL^−1^ penicillin – 100 μg mL^−1^ streptomycin. The RXR*α*-*bla* cells were seeded at 2000 in 6 μL assay medium per well in black-clear 1536-well tissue culture plates (Greiner Bio-One North America Inc.) using an FRD. After 5 h incubation of the assay plates at 37°C, 99% humidity, and 5% CO_2_, the positive control (9-*cis*-RA) along with DMSO-only wells and test compounds, were transferred to the assay plates at 23 nL using a Wako Pintool station. The assay plates were incubated with the compounds for 16 h at 37°C, 99% humidity, and 5% CO_2_. After addition of 1 μL LiveBLAzer^™^-FRET B/G (CCF4-AM) substrate (Thermo Fisher Scientific Inc.) using an FRD, the assay plates were incubated for 2 h at room temperature. Fluorescence signal (405 nm excitation, 460 and 530 nm emissions) in each well was measured using an Envision plate reader (PerkinElmer). The raw data were expressed as the ratio of 460/530 emissions.

### 2.6 | Data analysis

The experimental data (relative luminescence or fluorescence units) from each assay well were first normalized relative to the positive control (agonist assays: 100% and inhibition assays: −100%) and DMSO-only wells (0%) within each plate as follows: % Activation = [(V_compound_ − V_DMSO_)/ (V_pos_ − V_DMSO_)] *100, and % Inhibition = [(V_compound_ − V_DMSO_)/(V_DMSO_ − V_pos_)] *100, where Vcompound represents the compound well reads, V_pos_ and V_DMSO_ represents the average values of the positive control and DMSO-only wells respectively. The half maximum effective concentration (EC_50_) and half maximum inhibitory concentration (IC_50_), and maximum response values were obtained by fitting the concentration-response curves of each compound to a four-parameter Hill equation by nonlinear regression using GraphPad Prism version 9.0.0 (GraphPad Software, Boston, MA). AC_50_ (EC_50_ or IC_50_) is defined as the half maximum activity concentration.

## 3 | RESULTS

### 3.1 | Development of enzyme-based CYP26A1 inhibition assay

**FIGURE 2:**
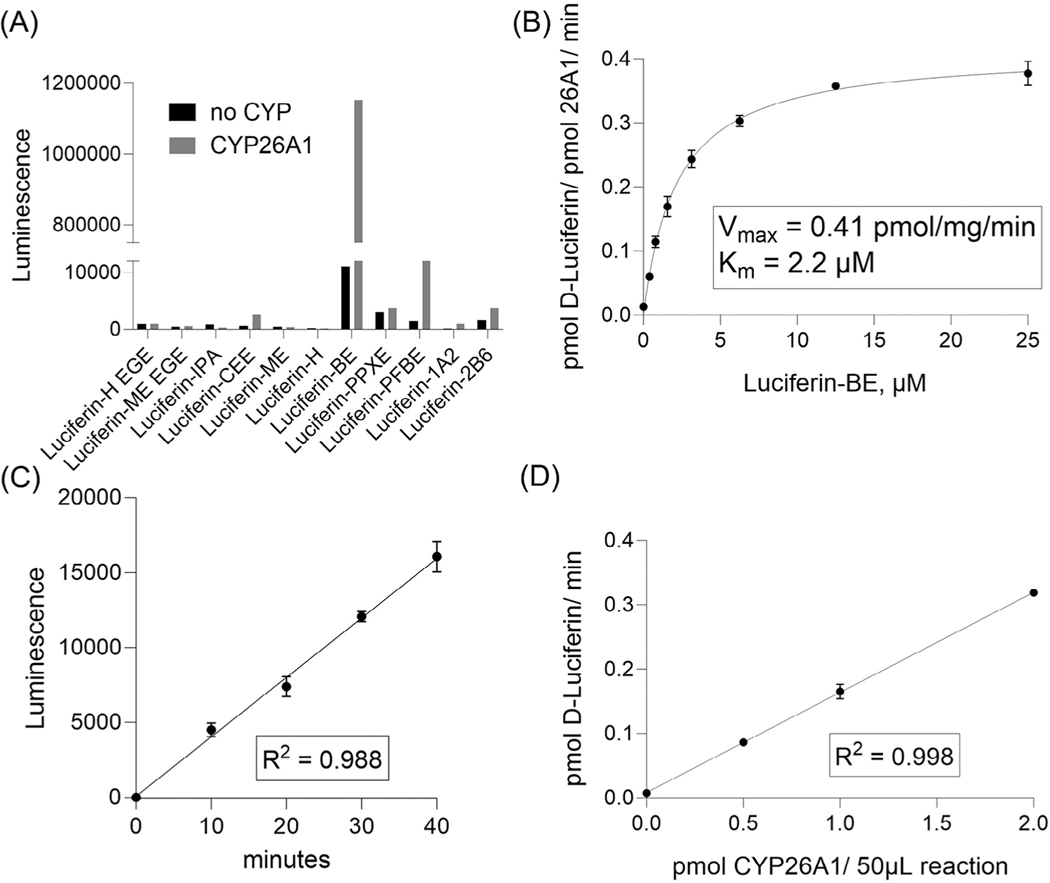
Development of CYP26A1 assay. (A) Luminogenic substrate reactivities with or without CYP26A1. (B) *V*_max_ and *K*_m_ determined for luciferin-BE. (C) Time course of the CYP26A1 reaction with 2 μM luciferin-BE. (D) The rates of luciferin-BE reaction at varied CYP26A1 enzyme amounts. Data shown for A are single determinants, data shown for B, C, and D are the mean ± SD from three experiments.

A P450-Glo^™^ assay technology has been used to develop enzyme-based CYP26A1 assays. In P450-Glo^™^ assay, CYP enzyme converts a luminogenic probe substrate to D-luciferin that is detected as light output from a second reaction with luciferase (Figure 1B). Light output is reduced by test compounds that inhibit the CYP enzyme. To find the optimal substrate for the assay, several luminogenic substrates^[23,25,26]^ were evaluated for their activities in the assay plus and minus a recombinant human CYP26A1 enzyme co-expressed with POR. Given the superficial structural similarities between retinoids and the D-luciferin derivatives (e.g., a COOH group on one end of the molecules at similar spacing from sites of CYP-dependent oxidation on the opposite end) along with known CYP26 substrate promiscuity, it was not unreasonable to expect to find a CYP26 luciferin derivative substrate.^[11,26]^ Among the substrates screened, the luciferin-BE substrate yielded the highest signal with CYP26A1 (Figure 2A). This identified luciferin-BE as an active CYP26A1 substrate with utility for identifying compounds that inhibit CYP26A1 activity.

To optimize the assay conditions of the luciferin-BE reaction with CYP26A1, seven concentrations of luciferin-BE substrate ranging from 0.39 to 25 μM plus a vehicle control were tested in a 96-well plate in triplicate, with 1.0 pmol CYP26A1 enzyme per 50 μL reaction volume for 30 min at 37°C. To derive reaction rates (pmol D-luciferin/pmol CYP26A1/min) from experimental data, relative luminescent units (RLU) were converted to CYP reaction product concentrations by interpolation from a D-luciferin standard curve (data not shown). Enzyme kinetic parameters (*K*_m_ and *V*_max_) were estimated through nonlinear regression analysis using the Michaelis-Menten model in GraphPad Prism (Figure 2B). Based on the determined *K*_m_ value, the luciferin-BE substrate concentration at 2 μM was selected for the assay. The assay was linear over time at 2 μM substrate for at least 40 min (linear regression: *R*^2^ = 0.988) (Figure 2C) with a close correlation between time and substrate conversion as indicated by a slope of 0.92 ± 0.08 from the log-log plot of time (10–40 min) versus net signal (data not shown). The assay showed a linear response (*R*^2^ = 0.998) with the increasing concentrations of CYP26A1, from 0.0 to 2.0 pmol per 50 μL reaction volume (Figure 2D). The final concentration of enzyme selected for the CYP26A1 assay was 1 pmol per 50 μL reaction volume.

### 3.2 | Optimization of CYP26A1 inhibition assay in a 1536-well plate

**FIGURE 3:**
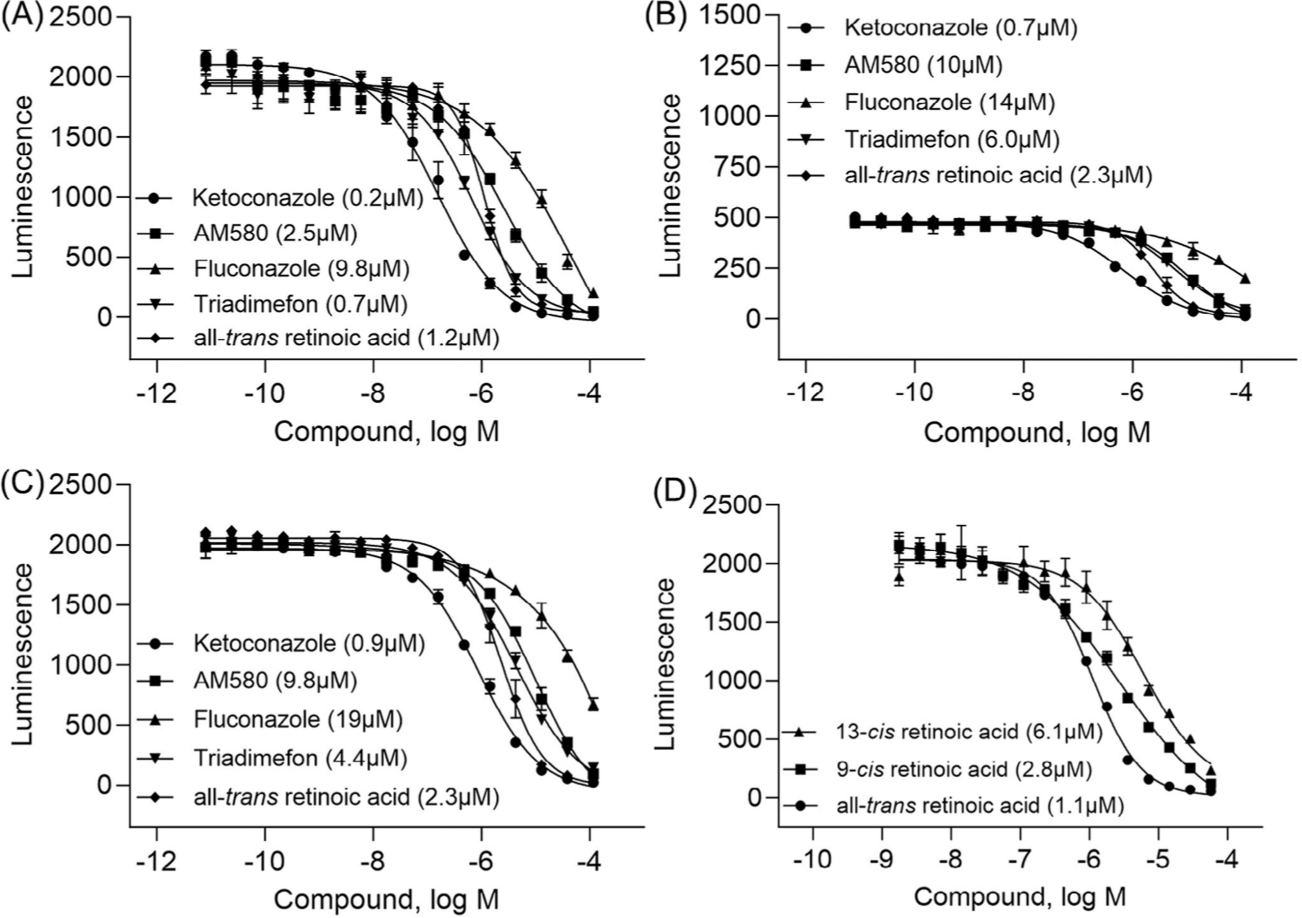
Concentration-response curves. Testing the positive control compounds in the CYP26A1 assay under various conditions, such as (A) 0.1% BSA + 0.025% CHAPS, (B) 0.1% BSA + 0.01% Triton X-100, and (C) 0.1% BSA + 0.01% Tween-20. (D) Comparison of the inhbitory effects of all-*trans* retinoic acid (atRA) with its isomers, including 9-*cis*-RA and 13-*cis*-RA with 0.1% BSA + 0.025% CHAPS condition. Each value represents the mean ± SD from three experiments.

The assay was miniaturized into 1536-well plates, with a final assay volume of 4 μL. To minimize well-to-well variations resulting from enzyme adhesion on the plate dispenser’s tubing, we assessed the impact of 0.1% Bovine Serum Albumin (BSA), along with various detergents such as triton X-100, tween-20, and CHAPS in our assay. The concentrations of triton X-100 and tween-20 were tested at 0.01%, a common concentration used in biochemical assays. Notably, the 0.025% CHAPS condition exhibited the highest potency, as indicated by the lowest IC_50_ values (Figure 3A–C). The assay was performed in three independent runs, and its performance was assessed using the following listed parameters, which are expressed as mean ± SD values from three runs. Signal to Background (S/B) ratios are 48.6 ± 2.4 and 25.7 ± 1.8 with atRA at 58 and 29 μM, respectively; coefficient of variation (CV) is 8.9% ± 2.6; Z′ is 0.7 ± 0.08. We validated additional isomers of atRA, including 9-*cis*-RA and 13-*cis*-RA, in our assay to assess the selectivity of these substrates for the CYP26A1 enzyme. The concentration-response curves representing the inhibitory effects of these three isomers are presented in Figure 3D. The IC_50_ values are: 1.1, 2.8, and 6.1 μM for atRA, 9-*cis*-RA, and 13-*cis*-RA, respectively. These inhibitory effects with 2- and 6-fold differences in IC_50_ values between atRA and its isomers, supports the fact that both these isomers still undergo phase I metabolism to several oxidized metabolites including 4-oxo-RA, 4-OH-RA, 18-OH-RA, and 5,6-epoxy-RA.^[3,27]^ The results further demonstrated that this assay is robust and suitable for high-throughput screening.

### 3.3 | Evaluation of CYP26A1 assay using a subset of compounds

**FIGURE 4:**
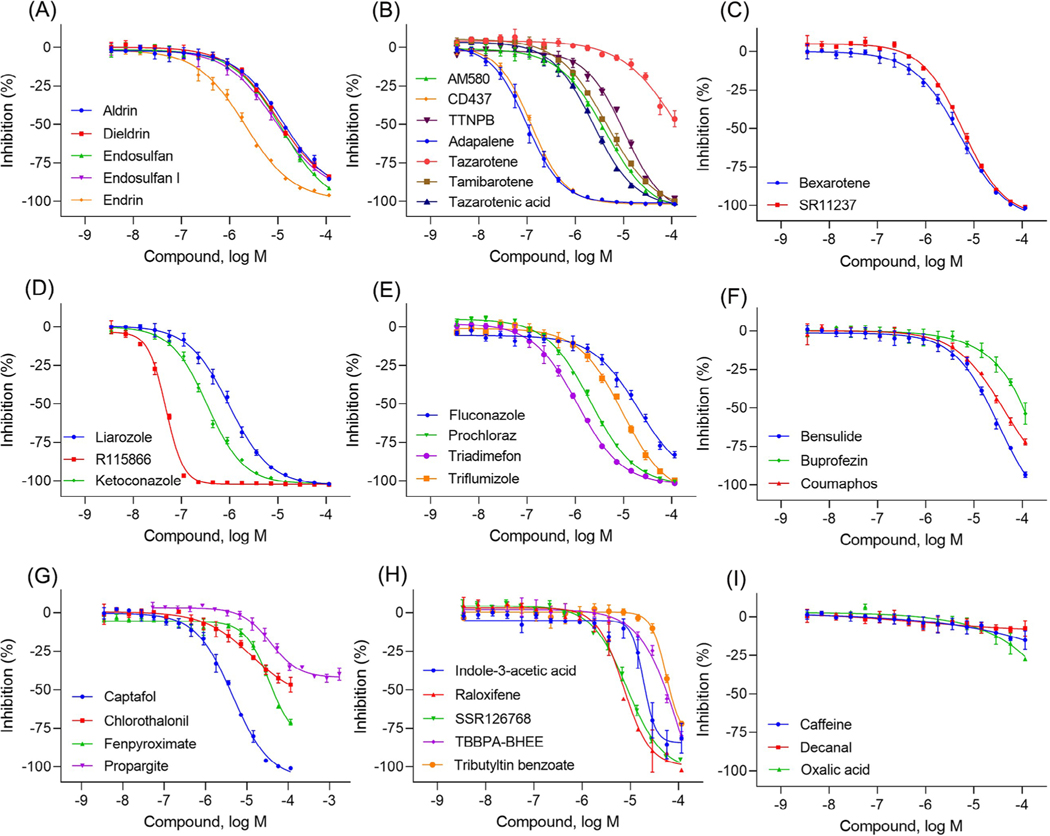
Concentration-response curves of the compounds tested in the CYP26A1 assay. (A) organochlorine pesticides: aldrin (IC_50_ = 12.8 μM), dieldrin (IC_50_ = 10 μM), endosulfan (IC_50_ = 12.1 μM), endosulfan I (IC_50_ = 9 μM), and endrin (IC_50_ = 2.1 μM); (B) retinoids: AM580 (IC_50_ = 4.1 μM), CD437 (IC_50_ = 0.1 μM), TTNPB (IC_50_ = 10.6 μM), adapalene (IC_50_ = 0.1 μM), tazarotene (IC_50_ = 54 μM), tamibarotene (IC_50_ = 4.5 μM), and tazarotenic acid (IC_50_ = 2.4 μM); (C) rexinoids: bexarotene (IC_50_ = 5.3 μM) and SR11237 (IC_50_ = 6.0 μM); (D) RAMBAs: ketoconazole (IC_50_ = 0.35 μM), liarozole (IC_50_ = 0.98 μM), and R115866 (IC_50_ = 0.05 μM); (E) conazole fungicides: fluconazole (IC_50_ = 20.2 μM), prochloraz (IC_50_ = 2.1 μM), triadimefon (IC_50_ = 1.1 μM), and triflumizole (IC_50_ = 9.5 μM); (F-H) diverse set of chemicals: bensulide (IC_50_ = 32.5 μM), buprofezin (IC_50_ = 80 μM), coumaphos (IC_50_ = 42.4 μM), captafol (IC_50_ = 4.6 μM), chlorothalonil (IC_50_ = 12.9 μM), fenpyroximate (IC_50_ = 34.8 μM), propargite (IC_50_ = 37.4 μM), indole-3-acetic acid (IC_50_ = 18.9 μM), raloxifene (IC_50_ = 7.2 μM), SSR126768 (IC_50_ = 8.6 μM), TBBPA-BHEE (IC_50_ = 119 μM), and tributyltin benzoate (IC_50_ = 55.5 μM); (I) negative controls. Each value represents the mean ± SD from three experiments.

To validate the CYP26A1 assay, a group of 39 compounds (Table 1) including known CYP26 and/or CYP inhibitors, retinoids and their analogues, and other diverse structural sets of compounds were selected. These compounds were previously predicted to be potential developmental toxicants associated with atRA signaling pathway disruptions.^[28,29]^ The assay was performed in three independent runs (*n* = 3). The highest concentration of each compound tested in the CYP26A1 assay was 115 μM, except for propargite (1.7 mM), and SSR126768 (109 μM). The compounds with maximum response greater than 40% are considered actives in the CYP26A1 assay. The concentration-response curves of the actives from the CYP26A1 assay are shown in Figure 4. The potencies (IC_50_) and maximum responses (%) of compounds tested in the CYP26A1 assay are given in Table 1. Thirty-three of the test compounds were shown to inhibit CYP26A1 enzymatic activity. The known CYP26A1 inhibitors, including AM580 (IC_50_ = 4.13 μM), bexarotene (IC_50_ = 5.3 μM), CD437 (IC_50_ = 0.12 μM), fluconazole (IC_50_ = 20.12 μM), liarozole (IC_50_ = 0.98 μM), ketoconazole (IC_50_ = 0.34 μM), R115866 (IC_50_ = 0.05 μM), raloxifene (IC_50_ = 11.2 μM), SR11237 (IC_50_ = 6.0 μM), tamibarotene (IC_50_ = 4.5 μM), tazarotenic acid (IC_50_ = 2.4 μM), and TTNPB (IC_50_ = 10.7 μM), were confirmed here. In contrast, three negative control compounds identified from the literature or other databases (caffeine, decanal, and oxalic acid) proved inactive. From this study, we identified several novel CYP26A1 inhibitors, such as, bensulide, chlorothalonil, coumaphos, fenpyroximate (Z,E), prochloraz, and SSR126768 (Figure 4) that have not been reported previously.

### 3.4 | Selectivity of CYP26A1 inhibitors in other relevant assays

**FIGURE 5:**
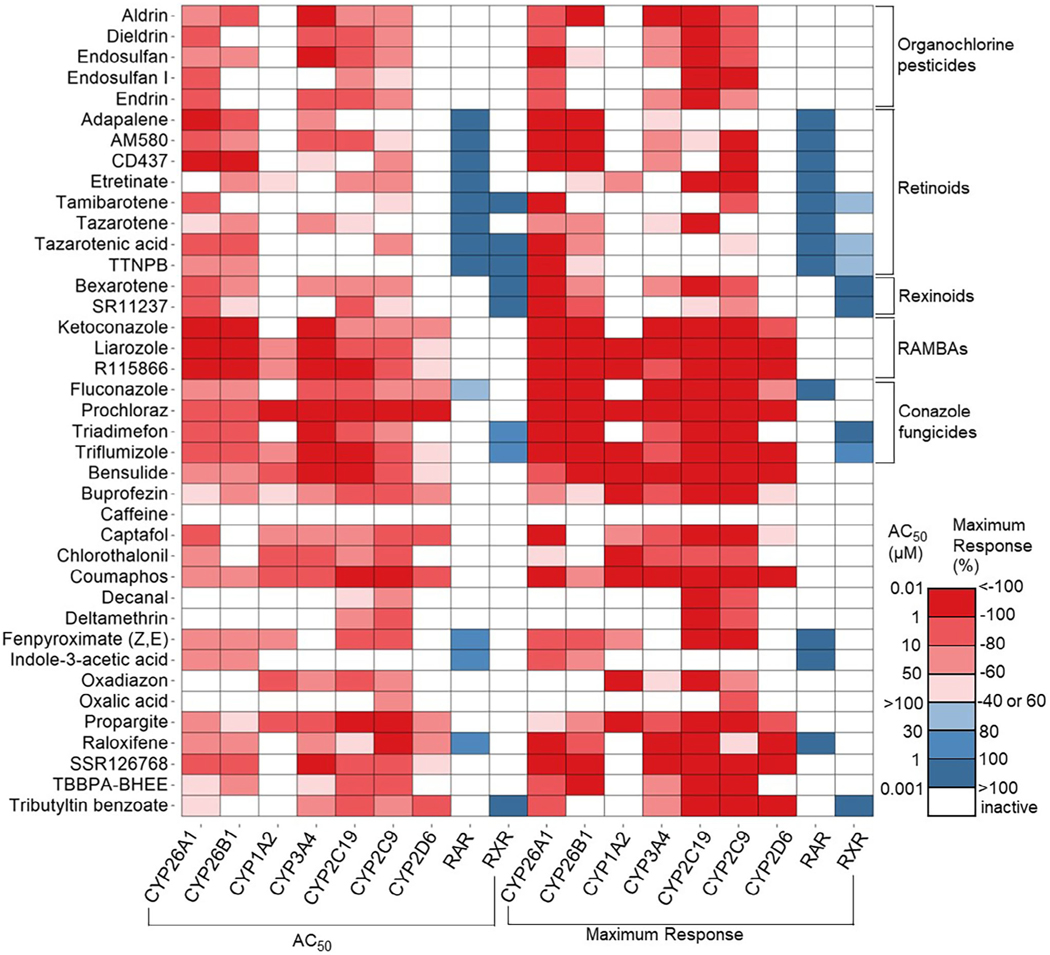
Compound activities in various assays. The heatmap is colored based on the half maximum activity, (AC_50_, μM) in the left portion and maximum response (%) values displayed on the right. Inhibitory assays include all CYPs, and the activation assays are RAR and RXR. The compounds are classified into different groups based on their chemical structures and functions, such as organochlorine pesticides and conazole fungicides, or their mechanism of action, including retinoids, rexinoids, and RAMBAs.

The selectivity toward CYP26A1 was examined against data from CYP26B1, CYP1A2, CYP3A4, CYP2C9, CYP2C19, CYP2D6, RAR, and RXR assays. In the CYP26B1 assay, the only pro-luminogenic substrate that exhibited activity with the enzyme is luciferin-BE, however with a significantly lower signal compared to CYP26A1. The IC_50_ values (μM) and maximum response (%) of the compounds in the CYP26B1, CYP1A2, CYP3A4, CYP2C9, CYP2C19, and CYP2D6 assays are shown in Figure 5. RAR and RXR transactivation reporter assays were included in the study to identify pathway agonists. The EC_50_ values (μM) and maximum response (%) of the compounds in these agonist assays are also shown in Figure 5. S/B ratios (mean ± SD, *n* = 3) obtained with their respective positive controls are: 1.7 ± 0.02 (atRA); 11.52 ± 0.6 (furafylline); 12.0 ± 2.3 (ketoconazole); 9.0 ± 0.8 (sulfaphenazole); 3.64 ± 0.3 (ketoconazole); 8.77 ± 0.4 (quinidine); 2.6 ± 0.3 (retinol); and 1.63 ± 0.1 (9-*cis* RA) from CYP26B1, CYP1A2, CYP3A4, CYP2C9, CYP2C19, CYP2D6, RAR, and RXR assays, respectively. S/B ratios for CYP26B1 and RXR assays are <2.0, but their overall assay performances were compensated by the lower CVs. Conazoles, including fluconazole, ketoconazole, prochloraz, R115866, triadimefon, and triflumizole, all demonstrated inhibitory effects in the CYP26A1 assay similar to their activity patterns on CYP26B1, CYP3A4, CYP2C9, and CYP2C19 (Figure 5). On the other hand, retinoids, including adapalene, AM580, CD437, etretinate, tamibrotene, tazarotene, tazarotenic acid, and TTNPB, were found to be more selective toward CYP26A1 and CYP26B1 (Figure 5). Although the CYP26B1 assay had a slightly lower signal window, it still produced some promising results with the compounds we evaluated in this study. The known atRA metabolic blockers (RAMBAs) ketoconazole, liarozole, and R115866 each demonstrated broad-spectrum inhibition across various CYP enzymes (Figure 5).

## 4 | DISCUSSION

In this study, we developed a novel enzyme-based CYP26A1 high-throughput screening assay and validated this assay using a diverse set of 39 chemicals. These chemicals were selected based on their demonstrated activity against diverse protein targets that are linked to the retinoid system, which contributes to developmental processes and toxicities.^[28,29]^ This test set included twelve known CYP26 inhibitors, various other CYP inhibitors, eight retinoids, two rexinoids (RXR-selective retinoids), and three RAMBAs. The assay performed well with S/B ratio exceeding 25-fold, CV of 8.9%, and a Z’ of 0.7. Of the 39 compounds tested here, 33 inhibited the CYP26A1 activity. Reliability of the assay for sensitive and specific detection of CYP26A1 inhibitors was demonstrated by observing inhibition by all 12 of the known CYP26A1 inhibitors tested (AM580, bexarotene, CD437, fluconazole, liarozole, ketoconazole, R115866, raloxifene, SR11237, tamibarotene, tazarotenic acid, and TTNPB), several novel inhibitors, such as, bensulide, chlorothalonil, coumaphos, fenpyroximate (Z,E), prochloraz, and SSR126768, and no effect from each negative control compound (caffeine, decanal, and oxalic acid). IC_50_ values from our assay for ketoconazole, liarozole, TTNPB, and bexarotene (0.34, 0.98, 10.7, and 5.3 μM, respectively) were in close correlation to the previously reported values of 0.55, 2.1, 3.7, and 12.3 μM, respectively, from recombinant CYP26A1 assays that used RA substrates and HPLC or LC-MS analysis.^[27,30,31]^ In contrast, R115866 inhibition was 10 times less potent in the luciferin-BE assay compared to an assay that used atRA as the probe substrate (50 vs. 5 nM).^[27,32]^ Furthermore, the IC_50_ of 1.1 μM for atRA inhibition of the luciferin-BE reaction (Figure 3D) is higher than would be expected if the two substrates are perfectly exclusive in the CYP26A1 active site given that CYP26A1 atRA *K*_m_ values have been reported in a range of about 9 to 50 nM.^[11]^ In aggregate, these results are consistent with the previous observation that CYP26A1 inhibition is substrate dependent.^[31]^ For example, the fold IC_50_ differences from assays that used either 9-*cis*-RA or tazarotenic acid as CYP26A1 probe substrate, ranged from unity to from 3- to 10-fold.^[31]^ Nevertheless, the best fit of our atRA inhibition curve has a Hill slope of −1, which is consistent with simple, non-cooperative atRA and luciferin-BE binding at a single active site on CYP26A1. Substrate-dependent CYP enzyme inhibition is well known, CYP3A4 being the best studied example.^[33,34]^ Additionally, the performance metrics demonstrated the assay’s sensitivity, robustness, and suitability for high throughput screening. Therefore, the identification of CYP26A1 inhibitors through this assay from large chemical libraries represents a significant advancement in the field of developmental toxicity assessment.

In the next step, we aim to use this assay for screening the Tox21’s collection of approximately 10,000 chemicals to identify CYP26A1 inhibitors. This collection was established for the ToxCast/Tox21 program, a collaborative effort among federal agencies aimed at developing innovative methods for assessing the potential risks of approved drugs and environmental chemicals to human health.^[35,36]^ The Tox21 chemical library has been extensively screened against various cellular targets and pathways using in vitro cell- and biochemical-based assays on a fully automated high throughput platform to prioritize compounds for further toxicological evaluation.^[37]^ To date, the Tox21 assays have enabled high-throughput screenings for a panel of nuclear receptors, stress response pathways, GPCRs, various CYP biochemical activities, enzyme targets, developmental-related cellular pathways such as TGF-*β*, SHH/GLI1, and the retinol signaling pathway (RAR),^[36,38]^ although the CYP26 family is not currently part of this portfolio. The CYP26 family plays a pivotal role in maintaining proper atRA levels within tissues, with their expression believed to be autoregulated based on atRA concentrations.^[9]^ Dysregulation of atRA metabolism and CYP26A1 activity can have detrimental effects on normal embryonic development, leading to developmental abnormalities.^[4]^ In the context of regulatory developmental toxicity for drugs and chemicals, it remains unclear whether any observed developmental defects can be attributed exclusively to disruption of retinoid signaling.^[39]^ To support recommendations regarding assay development for the retinoid system and the use of resulting data in the regulatory context for developmental toxicity, a CYP26 HTS assay could be considered as a top priority for addressing missing information in the retinoid system for New Approach Methodologies (NAMs). Therefore, the development and validation of robust screening assays to identify CYP26A1 inhibitors is significant.

Here, we assessed the inhibitory capabilities of the chemicals for several other CYPs (e.g., CYP1A2, CYP2C9, CYP3A4, and CYP26B1) known to be involved in the in vitro metabolism of atRA.^[3]^ Although the CYP26B1 assay demonstrated a lower S/B ratio with positive control compared to the CYP26A1 assay, we evaluated selectivity toward screening for CYP26A1 inhibition. As might be anticipated in a cell-free bioassay, the active compounds identified in the CYP26A1 assay had efficacy across a wider spectrum of other CYP assays, including CYP3A4, CYP2C9, CYP2C19, and CYP26B1 (Figure 5). This finding supports the involvement of CYP26, CYP3A, and CYP2C families, which are commonly implicated in atRA metabolism via 4-hydroxylation.^[3,9]^ However, in P450-Glo^™^ assays, different CYP enzymes can react with more than one P450-Glo^™^ substrate. The selectivity of a particular P450-Glo^™^ substrate in each CYP assay has been demonstrated by showing the sensitivity of the reactions^[26]^ and selectivity of the respective reactions is further supported by their insensitivity to compounds that inhibit other CYPs.

In this study, the chemicals were evaluated and categorized based on their structure and mode of actions, as depicted in the heatmap (Figure 5). The chemical groups demonstrating high potency in the CYP26A1 and other CYP assays were ranked in descending order as follows: RAMBAs, conazole fungicides, organochlorine pesticides, rexinoids, and retinoids. RAMBAs specifically target the CYP enzymes responsible for mediating retinoic acid metabolism.^[17]^ Among the RAMBAs investigated in this study, R115866 (talarazole), liarozole, and ketoconazole exhibited significant potency in both CYP26A1/B1 and CYP3A4 assays. R115866 is widely recognized as a highly potent and specific inhibitor of human CYP26A1 and CYP26B1, and other known inhibitors of CYP26A1 include liarozole and ketoconazole were confirmed.^[10,17,40]^ Most compounds tested here displayed inhibitory activity across a wide range of CYP assays, except for retinoids. Conversely, retinoids demonstrated a higher level of selectivity by inhibiting CYP26A1 and CYP26B1 (Figure 5). These findings support that the inhibition of retinoic acid metabolism by RAMBAs has the potential to enhance the endogenous retinoic acid levels in vivo, while the inhibition of CYP26 by retinoids has the potential to elevate the atRA gene expression in vitro.^[32,41]^

In conclusion, we successfully developed a novel high-throughput CYP26A1 inhibition assay for identifying CYP26A1 inhibitors and validated its reliability using a diverse set of compounds. These findings clearly demonstrate the assay robustness and its suitability for high-throughput screening purposes. By elucidating the complexities of atRA metabolism and its regulation, this research could bridge the gaps in retinoid system, which can contribute to a more comprehensive understanding of developmental toxicology and the potential risks associated with chemical exposure.

## Figures and Tables

**TABLE 1:** A list of 39 compounds, their corresponding IC_50_ values and maximum responses (in parenthesis) obtained from CYP26A1 assay and their mode of action. IC_50_ and maximum response values were expressed as mean ± standard deviation from three experiments.

Chemical name (CASRN)	Structure	IC_50_, μM (Maximum response,%)	Mode of action
Adapalene (106685–40–9)	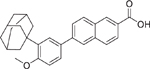	0.1 ± 0.0 (−101.03 ± 0.15)	A retinoid with higher affinity toward RAR-*β* and -*γ*.^[42]^
Aldrin (309–00–2)	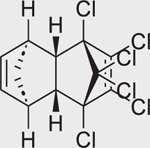	13.0 ± 1.8 (−95.35 ± 4.06)	An organochlorine pesticide with agonistic activity at RAR*β* and RAR*γ*.^[43]^
AM580 (102121–60–8)	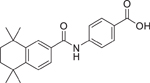	4.13 ± 0.21 (−107.4 ± 0.98)	A synthetic retinoid known for its selective activation of RAR-*α*^[44]^ and has been identified as a potential inhibitor of CYP26A1.^[27]^
Bensulide (741–58–2)	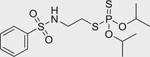	39.26 ± 15.74 (−93.3 ± 1.7)	An organophosphate herbicide that acts by inhibiting the plant cell division.^[45]^
Bexarotene (153559–49–0)	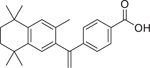	5.3 ± 0.6 (−110.13 ± 2.5)	A synthetic rexinoid known for its selective activation of RXR^[46]^ and identified as an inhibitor of both CYP26A1 and CYP26B1.^[30]^
Buprofezin (69327–76–0)	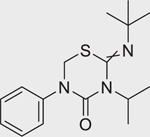	78.7 ± 38.6 (−75.7 ± 14)	A thiadiazine insecticide that inhibits chitin synthesis, which is a key component of insect cuticle.^[47]^
Caffeine (58–08–2)	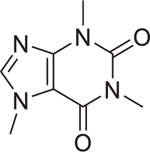	Inactive	A methylxanthine compound that binds to adenosine receptor and blocks the binding of adenosine to its receptor.^[48]^
Captafol (2425–06–1)	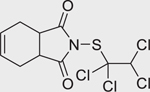	4.6 ± 0.07 (−108.27 ± 3.6)	A phthalimide class of fungicide that has exhibited endocrine-disrupting properties through the disruption of aromatase (CYP19A1) activity.^[49]^
CD437 (125316–60–1)	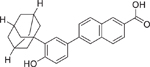	0.12 ± 0.01 (−101.8 ± 0.1)	A selective RAR-*γ* activator,^[44]^ this synthetic retinoid has demonstrated inhibitory effects on CYP26A1 and CYP26B1, utilizing tazarotenic acid as a substrate.^[31]^
Chlorothalonil (1897–45–6)	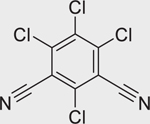	11.57 ± 3.76 (−55.26 ± 3.0)	An organochlorine fungicide that causes skeletal defects in animal models.^[50]^
Coumaphos (56–72–4)	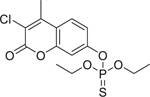	45.13 ± 8.76 (−105 ± 7.1)	An organophosphate insecticide that acts as an acetylcholinesterase inhibitor.^[51]^
Decanal (112–31–2)	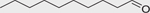	Inactive	A volatile aldehyde possessing antifungal activity.
Deltamethrin (52918–63–5)	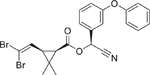	Inactive	A pyrethroid insecticide.
Dieldrin (60–57–1)	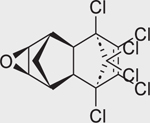	9.98 ± 0.92 (−93.07 ± 2.63)	An organochlorine pesticide with agonistic activity at RAR*β* and RAR*γ*.^[43]^
Endosulfan (115–29–7)	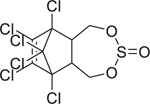	12.2 ± 0.8 (−104.2 ± 2.4)	An organochlorine pesticide with agonistic activity at RAR*β* and RAR*γ*^[43]^.
Endosulfan I (959–98–8)	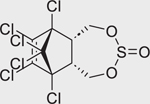	8.93 ± 1.07 (−94.61 ± 2.93)	An organochlorine pesticide and a stereoisomer of endosulfan II. Commercial endosulfan is a mixture of endosulfan I and endosulfan II isomers.^[52]^
Endrin (72–20–8)	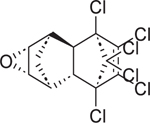	2.1 ± 0.2 (−99.11 ± 1.45)	An organochlorine pesticide with agonistic activity at RAR*β* and RAR*γ*.^[43]^
Etretinate (54350–48–0)	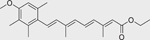	Inactive	An aromatic retinoid (second-generation) that exhibits anti-inflammatory activities.^[53]^
Fenpyroximate (Z,E) (111812–58–9)	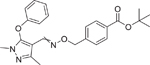	34.74 ± 1.62 (−83.43 ± 3.37)	A pyrazole acaricide that acts by inhibiting mitochondrial respiratory complex I in mites.^[54]^
Fluconazole (86386–73–4)	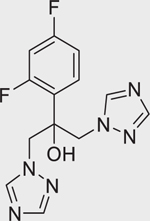	20.12 ± 2.85 (−100.33 ± 4.21)	A conazole fungicide that inhibits ergosterol synthesis and subsequently leads to the disruption of fungal membranes.^[55]^ It has been identified as a CYP26A1 inhibitor.^[27]^
Indole-3-acetic acid (87–51–4)	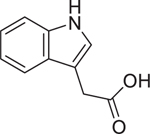	19.06 ± 2.35 (−85.0 ± 11.06)	A natural auxin-class phytohormone restrains budding yeast growth by inhibiting the target of rapamycin complex 1 (TORC1).^[56]^
Ketoconazole (65277–42–1)	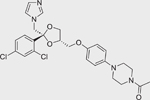	0.34 ± 0.03 (−101.8 ± 0.11)	A conazole fungicide that inhibits the biosynthesis of ergosterol, a vital component of fungal cell membrane.^[55]^ It is a potent inhibitor of human CYP3A isoforms^[57]^ and functions as a RAMBA by inhibiting CYP26 enzymes.^[27]^
Liarozole dihydrochloride (R-75251) 1883548–96–6	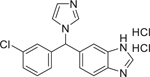	0.98 ± 0.14 (−102.7 ± 0.6)	An imidazole derivative that acts as a RAMBA by targeting all-*trans* retinoic acid 4-hydroxylases (CYP26 enzymes) for inhibition.^[17]^
Oxadiazon (19666–30–9)	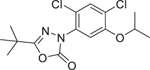	Inactive	An oxadiazole class of herbicide that inhibits protoporphyrinogen oxidase, a key enzyme for chlorophyll and heme biosynthesis.^[58]^
Oxalic acid (144–62–7)	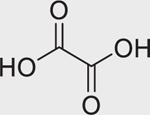	Inactive	An organic acid found naturally in plants.
Prochloraz (67747–09–5)	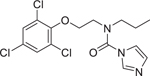	2.1 ± 0.12 (−102.9 ± 0.5)	An imidazole fungicide exhibiting endocrine disruptor activity.^[59]^
Propargite (2312–35–8)	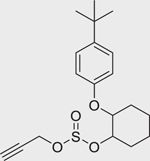	38.18 ± 14.2 (−41.98 ± 2.41)	An organosulfuracaricide that inhibits oxidative phosphorylation and disrupts ATP formation.^[60]^
R115866/Talarozole (870093–23–5)	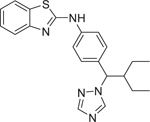	0.05 ± 0.004 (−102.13 ± 0.12)	A RAMBA that inhibits the all-*trans* retinoic acid 4-hydroxylases (CYP26A1 and CYP26B1).^[40]^
Raloxifene hydrochloride (82640–04–8)	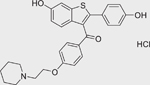	11.2 ± 6.32 (−102.3 ± 1.6)	An estrogen receptor modulator.^[61]^
SR11237 (146670–40–8)	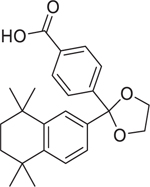	6.0 ± 0.16 (−107.5 ± 2.0)	A synthetic RXR specific agonist that is devoid of any RAR activity.^[62]^
SSR126768 (1437319–51–1)	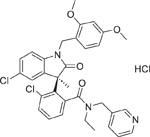	8.6 ± 0.78 (−102.3 ± 1.07)	A potent and highly selective oxytocin receptor antagonist.^[63]^
Tamibarotene/AM80 (94497–51–5)	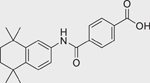	4.5 ± 0.11 (−105.5 ± 1.33)	A selective RAR-*α* activator,^[44]^ this synthetic retinoid has demonstrated inhibitory effects on CYP26A1 and CYP26B1, utilizing tazarotenic acid as a substrate.^[31]^
Tazarotene (118292–40–3)	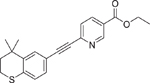	54.1 ± 6.1 (−71.1 ± 9.14)	An acetylenic retinoid that undergoes hydrolysis to form tazarotenic acid.^[64]^
Tazarotenic Acid (118292–41–4)	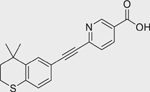	2.37 ± 0.1 (−103.3 ± 1.01)	An active form of tazarotene that binds to RAR-*β* and *-γ* and has been identified as a substrate of CYP26.^[64]^
TetrabromobisphenolA bis(2-hydroxyethyl) ether/TBBPA-BHEE (4162–45–2)	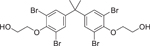	129.6 ± 43.7 (−79.8 ± 2.8)	A derivative of TBBPA, with potential neurotoxicity.^[65]^
Triadimefon (43121–43–3)	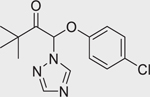	1.11 ± 0.05 (−102.4 ± 1.4)	A triazole fungicide that disrupts sterol production, the key constituents of fungal cell membranes.^[66]^
Tributyltin benzoate (4342–36–3)	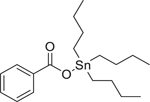	55.95 ± 4.66 (−81.74 ± 4.37)	An organotin derivative, which is an endocrine-disrupting chemical and activates RXRand PPAR-*γ*.^[67]^
Triflumizole (68694–11–1)	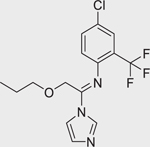	9.53 ± 0.71 (−109.8 ± 3.3)	A conazole fungicide that inhibits ergosterol biosynthesis.^[68]^
TTNPB/Arotinoid acid (71441–28–6)	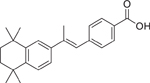	10.7 ± 1.0 (−108.9 ± 3.5)	A potent and selective synthetic retinoid that activates each RAR subtype and has been identified as a CYP26A1 inhibitor.^[27]^

## Data Availability

Data available on request from the authors.

## References

[1] Das BC., Thapa P., Kark R., Das S., Mahapatra S., Liu TC., Torregroza I., Wallac DP., Kambhampati S., Van Veldhuizen P., Verma A., Ray SK., & Evans T. (2014). Retinoic acid signaling pathways in development and diseases. Bioorganic & Medicinal Chemistry, 22(2), 673–683. 24393720 10.1016/j.bmc.2013.11.025 PMC4447240

[2] Bastien J, & Rochette-Egly C. (2004). Nuclear retinoid receptors and the transcription of retinoid-target genes. Gene, 328, 1–16. 15019979 10.1016/j.gene.2003.12.005

[3] Marill J, Idres N, Capron CC, Nguyen E, & Chabot GG (2003). Retinoic acid metabolism and mechanism of action: A review. Current Drug Metabolism, 4(1), 1–10. 12570742 10.2174/1389200033336900

[4] Cunningham TJ, & Duester G. (2015). Mechanisms of retinoic acid signalling and its roles in organ and limb development. Nature Reviews Molecular Cell Biology, 16(2), 110–123. 25560970 10.1038/nrm3932 PMC4636111

[5] McSorley LC, & Daly AK (2000). Identification of human cytochrome P450 isoforms that contribute to all-trans-retinoic acid 4-hydroxylation. Biochemical Pharmacology, 60(4), 517–526. 10874126 10.1016/s0006-2952(00)00356-7

[6] Nadin L, & Murray M. (1999). Participation of CYP2C8 in retinoic acid 4-hydroxylation in human hepatic microsomes. Biochemical Pharmacology, 58(7), 1201–1208. 10484078 10.1016/s0006-2952(99)00192-6

[7] Ross AC, & Zolfaghari R. (2011). Cytochrome P450s in the regulation of cellular retinoic acid metabolism. Annual Review of Nutrition, 31, 65–87. 10.1146/annurev-nutr-072610-145127 PMC3789243 21529158

[8] Zhong G, Ortiz D, Zelter A, Nath A, & Isoherranen N. (2018). CYP26C1 is a hydroxylase of multiple active retinoids and interacts with cellular retinoic acid binding proteins. Molecular Pharmacology, 93(5), 489–503. 29476041 10.1124/mol.117.111039 PMC5894800

[9] Thatcher JE, & Isoherranen N. (2009). The role of CYP26 enzymes in retinoic acid clearance. Expert Opinion on Drug Metabolism & Toxicology, 5(8), 875–886. 10.1517/17425250903032681 PMC2730205 19519282

[10] Nelson CH, Buttrick BR, & Isoherranen N. (2013). Therapeutic potential of the inhibition of the retinoic acid hydroxylases CYP26A1 and CYP26B1 by xenobiotics. Current Topics in Medicinal Chemistry, 13(12), 1402–1428. 23688132 10.2174/1568026611313120004 PMC4366427

[11] Isoherranen N, & Zhong G. (2019). Biochemical and physiological importance of the CYP26 retinoic acid hydroxylases. Pharmacology & Therapeutics, 204, 107400. 10.1016/j.pharmthera.2019.107400 PMC6881548 31419517

[12] Thatcher JE, Zelter A, & Isoherranen N. (2010). The relative importance of CYP26A1 in hepatic clearance of all-trans retinoic acid. Biochemical Pharmacology, 80(6), 903–912. 20513361 10.1016/j.bcp.2010.05.023 PMC2906711

[13] Snyder JM, Zhong G, Hogarth C, Huang W, Topping T, LaFrance J, Palau L, Czuba LC, Griswold M, Ghiaur G, & Isoherranen N. (2020). Knockout of Cyp26a1 and Cyp26b1 during postnatal life causes reduced lifespan, dermatitis, splenomegaly, and systemic inflammation in mice. The FASEB Journal, 34(12), 15788–15804. 33105029 10.1096/fj.202001734R PMC8139119

[14] Uehara M, Yashiro K, Mamiya S, Nishino J, Chambon P, Dolle P, & Sakai Y. (2007). CYP26A1 and CYP26C1 cooperatively regulate anterior-posterior patterning of the developing brain and the production of migratory cranial neural crest cells in the mouse. Developmental Biology, 302(2), 399–411. 17067568 10.1016/j.ydbio.2006.09.045

[15] Sirbu IO, Chis AR, & Moise AR (2020). Role of carotenoids and retinoids during heart development. BBA Molecular and Cell Biology of Lipids, 1865(11), 158636. 31978553 10.1016/j.bbalip.2020.158636 PMC7374046

[16] Stevison F, Jing J, Tripathy S, & Isoherranen N. (2015). Role of retinoic acid-metabolizing cytochrome P450s, CYP26, in inflammation and cancer. Advances in Pharmacology, 74, 373–412. 26233912 10.1016/bs.apha.2015.04.006 PMC4859867

[17] Njar VC, Gediya L, Purushottamachar P, Chopra P, Vasaitis TS, Khandelwal A, Mehta J, Huynh C, Belosay A, & Patel J. (2006). Retinoic acid metabolism blocking agents (RAMBAs) for treatment of cancer and dermatological diseases. Bioorganic & Medicinal Chemistry, 14(13), 4323–4340. 16530416 10.1016/j.bmc.2006.02.041

[18] Veith H, Southall N, Huang R, James T, Fayne D, Artemenko N, Shen M, Inglese J, Austin CP, Lloyd DG, & Auld DS (2009). Comprehensive characterization of cytochrome P450 isozyme selectivity across chemical libraries. Nature Biotechnology, 27(11), 1050–1055. 10.1038/nbt.1581 PMC2783980 19855396

[19] Wienkers LC, & Heath TG (2005). Predicting in vivo drug interactions from in vitro drug discovery data. Nature Reviews Drug Discovery, 4(10), 825–833. 16224454 10.1038/nrd1851

[20] Stresser DM, Turner SD, Blanchard AP, Miller VP, & Crespi CL (2002). Cytochrome P450 fluorometric substrates: Identification of isoform-selective probes for rat CYP2D2 and human CYP3A4. Drug Metabolism & Disposition, 30(7), 845–852. 12065444 10.1124/dmd.30.7.845

[21] Cali JJ, Klaubert DH, Daily WJ, Ho SKS, Frackman S, Hawkins EG, & Wood KV (2019). Luminescence-based methods and probes for measuring cytochrome p450 activity (U.S. Patent 10408819).

[22] Mazur CS, Kenneke JF, Goldsmith MR, & Brown C. (2009). Contrasting influence of NADPH and a NADPH-regenerating system on the metabolism of carbonyl-containing compounds in hepatic microsomes. Drug Metabolism and Disposition, 37(9), 1801–1805. 19541826 10.1124/dmd.109.027615

[23] Cali JJ, Ma D, Sobol M, Simpson DJ, Frackman S, Good TD, Daily WJ, & Liu D. (2006). Luminogenic cytochrome P450 assays. Expert Opinion on Drug Metabolism & Toxicology, 2(4), 629–645. 16859410 10.1517/17425255.2.4.629

[24] Chen Y, Sakamuru S, Huang R, Reese DH,&Xia,M.(2016).Identification of compounds that modulate retinol signaling using a cell-based qHTS assay. Toxicology in Vitro, 32, 287–296. 26820057 10.1016/j.tiv.2016.01.011 PMC4779714

[25] Ugalde SO, Ma DP, Cali JJ, & Commandeur JNM (2019). Evaluation of luminogenic substrates as probe substrates for bacterial cytochrome P450 enzymes: Application to *Mycobacterium tuberculosis*. SLAS Discovery, 24(7), 745–754. 31208248 10.1177/2472555219853220 PMC6651611

[26] Cali JJ, Ma D, Wood MG, Meisenheimer PL, & Klaubert DH (2012). Bioluminescent assays for ADME evaluation: Dialing in CYP selectivity with luminogenic substrates. Expert Opinion on Drug Metabolism & Toxicology, 8(9), 1115–1130. 22686499 10.1517/17425255.2012.695345

[27] Thatcher JE, Buttrick B, Shaffer SA, Shimshoni JA, Goodlett DR, Nelson WL, & Isoherranen N. (2011). Substrate specificity and ligand interactions of CYP26A1, the human liver retinoic acid hydroxylase. Molecular Pharmacology, 80(2), 228–239. 21521770 10.1124/mol.111.072413 PMC3141886

[28] Baker NC, Pierro JD, Taylor LW, & Knudsen TB (2023). Identifying candidate reference chemicals for in vitro testing of the retinoid pathway for predictive developmental toxicity. Altex, 40(2), 217–236. 35796328 10.14573/altex.2202231 PMC10765368

[29] Pierro JD, Ahir BK, Baker NC, Kleinstreuer NC, Xia M, & Knudsen TB (2022). Computational model for fetal skeletal defects potentially linked to disruption of retinoic acid signaling. Frontiers in Pharmacology, 13, 971296. 36172177 10.3389/fphar.2022.971296 PMC9511990

[30] Diaz P, Huang W, Keyari CM, Buttrick B, Price L, Guilloteau N, Tripathy S, Sperandio VG, Fronczek FR, Astruc-Diaz F, & Isoherranen N. (2016). Development and characterization of novel and selective inhibitors of cytochrome P450 CYP26A1, the human liver retinoic acid hydroxylase. Journal of Medicinal Chemistry, 59(6), 2579–2595. 26918322 10.1021/acs.jmedchem.5b01780 PMC4836378

[31] Foti RS, Diaz P, & Douguet D. (2016). Comparison of the ligand binding site of CYP2C8 with CYP26A1 and CYP26B1: A structural basis for the identification of new inhibitors of the retinoic acid hydroxylases. Journal of Enzyme Inhibition and Medicinal Chemistry, 31(sup2), 148–161. 10.1080/14756366.2016.1193734 PMC6628712 27424662

[32] Stoppie P, Borgers M, Borghgraef P, Dillen L, Goossens J, Sanz G, Szel H, Van Hove C, Van Nyen G, Nobels G, Vanden Bossche H, Venet M, Willemsens G, & Van Wauwe J. (2000). R115866 inhibits all-trans-retinoic acid metabolism and exerts retinoidal effects in rodents. The Journal of Pharmacology and Experimental Therapeutics, 293(1), 304–312. 10734183

[33] Wang RW, Newton DJ, Liu N, Atkins WM, & Lu AY (2000). Human cytochrome P-450 3A4: In vitro drug-drug interaction patterns are substrate-dependent, Drug Metabolism and Disposition, 28(3), 360–366. 10681383

[34] Ekroos M, & Sjogren T. (2006). Structural basis for ligand promiscuity in cytochrome P450 3A4. PNAS, 103(37), 13682–13687. 16954191 10.1073/pnas.0603236103 PMC1564212

[35] Tice RR, Austin CP, Kavlock RJ, & Bucher JR (2013). Improving the human hazard characterization of chemicals: A Tox21 update. Environmental Health Perspectives, 121(7), 756–765. 23603828 10.1289/ehp.1205784 PMC3701992

[36] Lynch C, Sakamuru S, Ooka M, Huang R, Klumpp-Thomas C, Shinn P, Gerhold D, Rossoshek A, Michael S, Casey W, Santillo MF, Fitzpatrick S, Thomas RS, Simeonov A, & Xia M. (2023). High-throughput screening to advance in vitro toxicology: Accomplishments, challenges, and future directions. Annual Review of Pharmacology and Toxicology, 64, 191–209. 10.1146/annurev-pharmtox-112122-104310 PMC10822017 37506331

[37] Attene-Ramos MS, Miller N, Huang R, Michael S, Itkin M, Kavlock RJ, Austin CP, Shinn P, Simeonov A, Tice RR, & Xia M. (2013). The Tox21 robotic platform for the assessment of environmental chemicals–from vision to reality. Drug Discovery Today, 18(15–16), 716–723. 23732176 10.1016/j.drudis.2013.05.015 PMC3771082

[38] Sakamuru S, Huang R, & Xia M. (2022). Use of Tox21 screening data to evaluate the COVID-19 drug candidates for their potential toxic effects and related pathways. Frontiers in Pharmacology, 13, 935399. 35910344 10.3389/fphar.2022.935399 PMC9333127

[39] Knudsen TB, Pierro JD, & Baker NC (2021). Retinoid signaling in skeletal development: Scoping the system for predictive toxicology. Reproductive Toxicology, 99, 109–130. 33202217 10.1016/j.reprotox.2020.10.014 PMC11451096

[40] Stevison F, Hogarth C, Tripathy S, Kent T, & Isoherranen N. (2017). Inhibition of the all-trans Retinoic Acid (atRA) hydroxylases CYP26A1 and CYP26B1 results in dynamic, tissue-specific changes in endogenous atRA signaling. Drug Metabolism & Disposition, 45(7), 846–854. 28446509 10.1124/dmd.117.075341 PMC5469401

[41] Gomaa MS, Armstrong JL, Bobillon B, Veal GJ, Brancale A, Redfern CP, & Simons C. (2008). Novel azolyl-(phenylmethyl)]aryl/heteroarylamines: Potent CYP26 inhibitors and enhancers of all-trans retinoic acid activity in neuroblastoma cells. Bioorganic & Medicinal Chemistry, 16(17), 8301–8313. 18722776 10.1016/j.bmc.2007.06.048

[42] Piskin S, & Uzunali E. (2007). A review of the use of adapalene for the treatment of acne vulgaris. Therapeutics and Clinical Risk Management, 3(4), 621–624. 18472984 PMC2374937

[43] Lemaire G, Balaguer P, Michel S, & Rahmani R. (2005). Activation of retinoic acid receptor-dependent transcription by organochlorine pesticides. Toxicology and Applied Pharmacology, 202(1), 38–49. 15589975 10.1016/j.taap.2004.06.004

[44] Schneider SM, Offterdinger M, Huber H, & Grunt TW (2000). Activation of retinoic acid receptor alpha is sufficient for full induction of retinoid responses in SK-BR-3 and T47D human breast cancer cells. Cancer Research, 60(19), 5479–5487. 11034091

[45] Zilkah S, Osband ME, & McCaffrey R. (1981). Effect of inhibitors of plant cell division on mammalian tumor cells in vitro. Cancer Research, 41(5), 1879–1883. 7214355

[46] Rigas JR, & Dragnev KH (2005). Emerging role of rexinoids in non-small cell lung cancer: Focus on bexarotene. The Oncologist, 10(1), 22–33. 15632250 10.1634/theoncologist.10-1-22

[47] Chang X, Yuan Y, Zhang T, Wang D, Du X, Wu X, Chen H, Chen Y, Jiao Y, & Teng H. (2015). The toxicity and detoxifying mechanism of cycloxaprid and buprofezin in controlling *Sogatella furcifera* (Homoptera: Delphacidae). Journal of Insect Science (Online), 15(1), 98. 26175461 10.1093/jisesa/iev077 PMC4677492

[48] Fiani B, Zhu L, Musch BL, Briceno S, Andel R, Sadeq N, & Ansari AZ (2021). The neurophysiology of caffeine as a central nervous system stimulant and the resultant effects on cognitive function. Cureus, 13(5), e15032. 34150383 10.7759/cureus.15032 PMC8202818

[49] Ge H, Chen L, Su Y, Jin C, & Ge RS (2018). Effects of folpet, captan, and captafol on human aromatase in JEG-3 cells. Pharmacology, 102(12), 81–87. 29953993 10.1159/000484171

[50] Silva JND, Monteiro NR, Antunes PA, & Favareto APA (2020). Maternal and developmental toxicity after exposure to formulation of chlorothalonil and thiophanate-methyl during organogenesis in rats. Anais da Academia Brasileira de Ciencias, 92(4), e20191026. 33206784 10.1590/0001-3765202020191026

[51] Williamson SM, Moffat C, Gomersall MA, Saranzewa N, Connolly CN, & Wright GA (2013). Exposure to acetylcholinesterase inhibitors alters the physiology and motor function of honeybees. Frontiers in Physiology, 4, 13. 23386834 10.3389/fphys.2013.00013 PMC3564010

[52] Bussian BM, Pandelova M, Lehnik-Habrink P, Aichner B, Henkelmann B, & Schramm KW (2015). Persistent endosulfan sulfate is found with highest abundance among endosulfan I, II, and sulfate in German forest soils. Environmental Pollution, 206, 661–666. 26319511 10.1016/j.envpol.2015.08.023

[53] Ward A, Brogden RN, Heel RC, Speight TM, & Avery GS (1983). Etretinate. A review of its pharmacological properties and therapeutic efficacy in psoriasis and other skin disorders. Drugs, 26(1), 9–43. 6224672 10.2165/00003495-198326010-00002

[54] Lee HY, Lee BK, Jeung KW, Lee GS, Jung YH, & Jeong IS (2012). A case of near-fatal fenpyroximate intoxication: The role of percutaneous cardiopulmonary support and therapeutic hypothermia. Clinical Toxicology (Philadelphia, Pa.), 50(9), 858–861. 22963274 10.3109/15563650.2012.720987

[55] Spampinato C, & Leonardi D. (2013). Candida infections, causes, targets, and resistance mechanisms: Traditional and alternative antifungal agents. BioMed Research International, 2013, 204237. 23878798 10.1155/2013/204237 PMC3708393

[56] Nicastro R, Raucci S, Michel AH, Stumpe M, Osuna GMG, Jaquenoud M, Kornmann B, & De Virgilio C. (2021). Indole-3-acetic acid is a physiological inhibitor of TORC1 in yeast. Plos Genetics, 17(3), e1009414. 33690632 10.1371/journal.pgen.1009414 PMC7978357

[57] Greenblatt DJ, Zhao Y, Venkatakrishnan K, Duan SX, Harmatz JS, Parent SJ, Court MH, & von Moltke LL (2011). Mechanism of cytochrome P450–3A inhibition by ketoconazole. Journal of Pharmacy and Pharmacology, 63(2), 214–221. 21235585 10.1111/j.2042-7158.2010.01202.x

[58] Sherwani SI (2015). Modes of action of different classes of herbicides. IntechOpen, sine loco.

[59] Vinggaard AM, Hass U, Dalgaard M, Andersen HR, Bonefeld-Jorgensen E, Christiansen S, Laier P, & Poulsen ME (2006). Prochloraz: An imidazole fungicide with multiple mechanisms of action. International Journal of Andrology, 29(1), 186–192. 16466539 10.1111/j.1365-2605.2005.00604.x

[60] Pridgeon JW, Pereira RM, Becnel JJ, Allan SA, Clark GG, & Linthicum KJ (2008). Susceptibility of *Aedes aegypti, Culex quinquefasciatus* Say, and *Anopheles quadrimaculatus* Say to 19 pesticides with different modes of action. Journal of Medical Entomology, 45(1), 82–87. 18283946 10.1603/0022-2585(2008)45[82:soaacq]2.0.co;2

[61] Gizzo S, Saccardi C, Patrelli TS, Berretta R, Capobianco G, Di Gangi S, Vacilotto A, Bertocco A, Noventa M, Ancona E, D’Antona D, & Nardelli GB (2013). Update on raloxifene: Mechanism of action, clinical efficacy, adverse effects, and contraindications. Obstetrical & Gynecological Survey, 68(6), 467–481. 23942473 10.1097/OGX.0b013e31828baef9

[62] Benoit G, Altucci L, Flexor M, Ruchaud S, Lillehaug J, Raffelsberger W, Gronemeyer H, & Lanotte M. (1999). RAR-independent RXR signaling induces t(15;17) leukemia cell maturation. The EMBO Journal, 18(24), 7011–7018. 10601023 10.1093/emboj/18.24.7011 PMC1171764

[63] Serradeil-Le Gal C, Valette G, Foulon L, Germain G, Advenier C, Naline E, Bardou M, Martinolle JP, Pouzet B, Raufaste D, Garcia C, Double-Cazanave E, Pauly M, Pascal M, Barbier A, Scatton B, Maffrand JP, & Le Fur G. (2004). SSR126768A (4-chloro-3-[(3R-+)-5-chloro-1-(2,4-dimethoxybenzyl)-3-methyl-2-oxo-2,3-dihydro-1H-indol-3-yl]-N-ethyl-N-(3-pyridylmethyl)-benzamide, hydrochloride): A new selective and orally active oxytocin receptor antagonist for the prevention of preterm labor. Journal of Pharmacology and Experimental Therapeutics, 309(1), 414–424. 14722330 10.1124/jpet.103.061200

[64] Foti RS, Isoherranen N, Zelter A, Dickmann LJ, Buttrick BR, Diaz P, & Douguet D. (2016). Identification of tazarotenic acid as the first xenobiotic substrate of human retinoic acid hydroxylase CYP26A1 and CYP26B1. The Journal of Pharmacology and Experimental Therapeutics, 357(2), 281–292. 26937021 10.1124/jpet.116.232637 PMC4851321

[65] Liu QS, Liu N, Sun ZD, Zhou QF, & Jiang GB (2018). Intranasal administration of tetrabromobisphenol A bis(2-hydroxyethyl ether) induces neurobehavioral changes in neonatal Sprague Dawley rats. Journal of Environmental Sciences-China, 63, 76–86. 29406119 10.1016/j.jes.2017.05.036

[66] Thabit TMA, Abdelkareem EM, Bouqellah NA, & Shokr SA (2021). Triazole fungicide residues and their inhibitory effect on some trichothecenes mycotoxin excretion in wheat grains. Molecules (Basel, Switzerland), 26(6), 1784. 33810162 10.3390/molecules26061784 PMC8005144

[67] Grun F, Watanabe H, Zamanian Z, Maeda L, Arima K, Cubacha R, Gardiner DM, Kanno J, Iguchi T, & Blumberg B. (2006). Endocrine-disrupting organotin compounds are potent inducers of adipogenesis in vertebrates. Molecular Endocrinology, 20(9), 2141–24455. 16613991 10.1210/me.2005-0367

[68] Xi J, Shao J, Wang Y, Wang X, Yang H, Zhang X, & Xiong D. (2019). Acute toxicity of triflumizole to freshwater green algae *Chlorella vulgaris*. Pesticide Biochemistry and Physiology, 158, 135–142. 31378349 10.1016/j.pestbp.2019.05.002

